# The Genetic Architecture of Degenerin/Epithelial Sodium Channels in *Drosophila*

**DOI:** 10.1534/g3.112.005272

**Published:** 2013-03-01

**Authors:** Kathleen M. Zelle, Beika Lu, Sarah C. Pyfrom, Yehuda Ben-Shahar

**Affiliations:** Department of Biology, Washington University in St. Louis, St. Louis, Missouri 63130

**Keywords:** Degenerin/epithelial sodium channel, chemosensation, mechanosensation, phylogeny, fruit fly

## Abstract

Degenerin/epithelial sodium channels (DEG/ENaC) represent a large family of animal-specific membrane proteins. Although the physiological functions of most family members are not known, some have been shown to act as nonvoltage gated, amiloride-sensitive sodium channels. The DEG/ENaC family is exceptionally large in genomes of *Drosophila* species relative to vertebrates and other insects. To elucidate the evolutionary history of the DEG/ENaC family in *Drosophila*, we took advantage of the genomic and genetic information available for 12 *Drosophila* species that represent all the major species groups in the *Drosophila* clade. We have identified 31 family members (termed *pickpocket* genes) in *Drosophila melanogaster*, which can be divided into six subfamilies, which are represented in all 12 species. Structure prediction analyses suggested that some subunits evolved unique structural features in the large extracellular domain, possibly supporting mechanosensory functions. This finding is further supported by experimental data that show that both *ppk1* and *ppk26* are expressed in multidendritic neurons, which can sense mechanical nociceptive stimuli in larvae. We also identified representative genes from five of the six DEG/ENaC subfamilies in a mosquito genome, suggesting that the core DEG/ENaC subfamilies were already present early in the dipteran radiation. Spatial and temporal analyses of expression patterns of the various *pickpocket* genes indicated that paralogous genes often show very different expression patterns, possibly indicating that gene duplication events have led to new physiological or cellular functions rather than redundancy. In summary, our analyses support a rapid early diversification of the DEG/ENaC family in Diptera followed by physiological and/or cellular specialization. Some members of the family may have diversified to support the physiological functions of a yet unknown class of ligands.

All cells use a complex array of ion channels to maintain the appropriate ionic gradients across membrane barriers, including the plasma membrane and intracellular compartments and organelles. One enigmatic group of ion channels is the Degenerin/epithelial Na^+^ channel (DEG/ENaC) family. Although the physiological functions of most family members are not well understood, at least some members seem to act as nonvoltage-gated, amiloride-sensitive sodium channels (Bianchi and Driscoll 2002; Garty and Palmer 1997). Various natural ligands and mechanical stimuli can activate or modulate channel functions. These include the neuropeptides FMRFamide (Askwith *et al.* 2000; Durrnagel *et al.* 2010; Golubovic *et al.* 2007; Green *et al.* 1994; Kellenberger and Schild 2002; Lingueglia *et al.* 1995; Xie *et al.* 2003), FFamide, SFamide (Deval *et al.* 2003; Sherwood and Askwith, 2008, 2009), and dynorphin-related opioid peptides (Sherwood and Askwith 2009). In addition, some mammalian family members are gated by extracellular protons (Benson *et al.* 2002; Price *et al.* 2001; Waldmann *et al.* 1997; Xie *et al.* 2003; Xiong *et al.* 2004). Recently, several sulfhydryl compounds (Cho and Askwith 2007) and small polyamines such as agmatine (Yu *et al.* 2010) also were shown to modulate the channel functions of specific mammalian family members. Finally, data also support a role for specific DEG/ENaC subunits in pheromone-dependent behaviors as well as in chemosensory functions underlying male courtship behaviors in *Drosophila* (Ben-Shahar 2011; Ben-Shahar *et al.* 2007; 2010; Lin *et al.* 2005; Lu *et al.* 2012; Starostina *et al.* 2012; Thistle *et al.* 2012; Toda *et al.* 2012).

DEG/ENaC family members also have been implicated in mechanosensation in *Caenorhabditis elegans*, mammals, and *Drosophila* (Arnadottir *et al.* 2011; Bazopoulou *et al.* 2007; Geffeney *et al.* 2011; Lu *et al.* 2009; O’Hagan *et al.* 2005; Price *et al.* 2001; Simon *et al.* 2010; Tsubouchi *et al.* 2012; Zhang *et al.* 2004; Zhong *et al.* 2010). Together, these data indicate that DEG/ENaC channels have evolved to serve many different physiological functions, acting as ionotropic receptors to diverse extracellular stimuli.

Functional and structural studies of DEG/ENaC channels demonstrated that channels are likely hetero or homotrimeric (Benson *et al.* 2002; Canessa *et al.* 1994; Eskandari *et al.* 1999; Jasti *et al.* 2007; Kellenberger and Schild 2002; Zha *et al.* 2009b). Electrophysiological studies indicated that subunit composition has a significant effect on the pharmacological and kinetic properties of assembled channels, suggesting that channel subunit composition plays a critical regulatory mechanism (Askwith *et al.* 2004; Benson *et al.* 2002; Chu *et al.* 2004; Xie *et al.* 2003; Zha *et al.* 2009a; Zhang *et al.* 2008). Hence, channel subunit diversity in a single animal is likely to represent diversity in activating stimuli and/or complex channel regulation.

Although the DEG/ENaC family is highly diverse across animalia, all family members share several highly conserved structural and topological features (Bianchi 2007; Bianchi and Driscoll 2002; Corey and Garcia-Anoveros 1996; Tavernarakis and Driscoll, 2000, 2001). Conserved topologies include two transmembrane helixes, two short intracellular domains, and a large cysteine-rich extracellular loop (Figure 1) (Ben-Shahar 2011).

**Figure 1:**
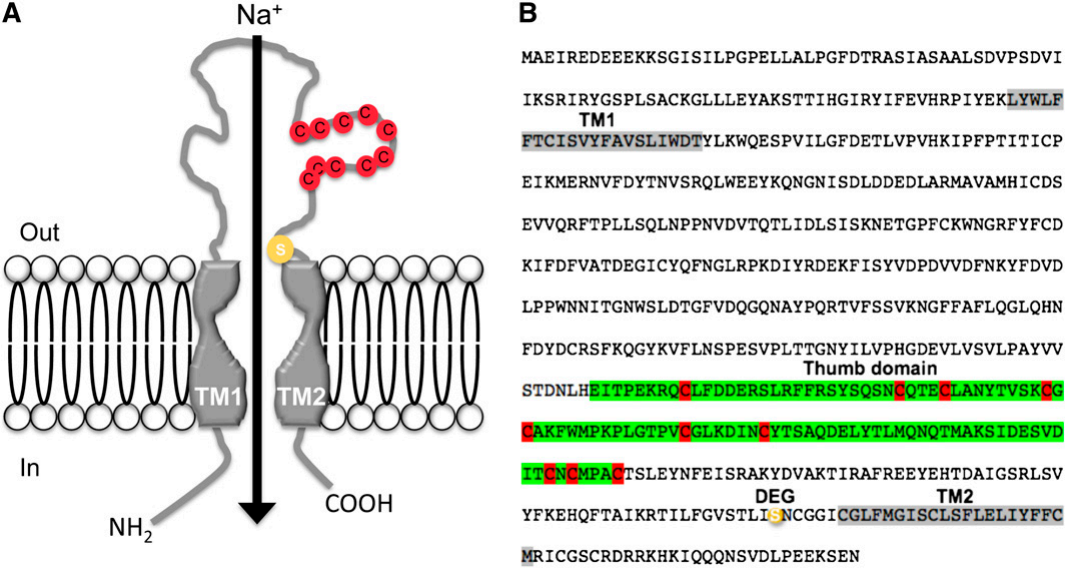
(A) Illustration depicting a typical DEG/ENaC subunit. TM, transmembrane domain; Red circles represent conserved cysteines; yellow circle represents the “DEG” residue, which in some subunits results in a constitutively open channel state when mutated (Adams *et al.* 1998; Kellenberger *et al.* 2002; Snyder *et al.* 1998, 2000). (B) The protein sequence of PPK, one of the first DEG/ENaC subunits that was identified in the *Drosophila* genome (Adams *et al.* 1998). Alignment of all the *Drosophila* subunits described in Table 1 and Table S1 indicate the presence of a highly conserved cysteine-enriched domain (also see Figure 7A, thumb domain), highlighted in green. Conserved cysteines are highlighted in red; DEG, a predicted “deg” residue, is highlighted in yellow. TM1 and TM2 represent the predicted transmembrane domains 1 and 2, respectively.

Surprisingly, mammalian genomes encode only eight to nine independent DEG/ENaC subunits, whereas the genomes of the worm *C. elegans* and various *Drosophila* species harbor a significantly larger number of DEG/ENaC-like genes [31 in *Drosophila melanogaster* and 30 in *C. elegans* (Bazopoulou *et al.* 2007; Ben-Shahar 2011; Liu *et al.* 2003a; Liu *et al.* 2003b; Studer *et al.* 2011)]. Consequently, DEG/ENaC genes represent one of the largest ion channel families in the *Drosophila* genome. The high diversification of DEG/ENaC protein sequences across distant animal species makes it difficult to evaluate whether the family expanded in some invertebrate species or whether it contracted in vertebrates. Nevertheless, the remarkable diversity of *ppk* genes in *Drosophila* suggests two alternative hypotheses. The first would suggest DEG/ENaC ion channels serve a wider range of physiological functions relative to their roles in mammals. An alternative hypothesis would be that DEG/ENaC channels in *Drosophila* evolved to serve highly specialized functions, predicting that each specific DEG/ENaC channel type in flies is responsible for a narrow slice of the physiological functions performed by a mammalian family member. However, identifying physiological and functional homology between family members across distant species is often impossible due to the poor overall protein sequence conservation of the extracellular loop domains. Thus, protein alignment analyses alone are typically not sufficient to draw physiological homology conclusions. Consequently, newly identified family members typically require physiological analyses *de novo*.

The increasing interest in DEG/ENaC-dependent signaling, their emerging importance in diverse physiological functions, and their high variability across different animal genomes suggests these ion channels may have played an important role in animal evolution. Here we reason that the dramatic diversity of the DEG/ENaC family in the *Drosophila* lineage represents an excellent opportunity to use evolutionary and molecular studies to gain new insights into the possible unique role of these channels in diverse physiological systems in general and insect biology in particular.

## Materials and Methods

### Phylogenetic analyses

*Drosophila melanogaster ppk* family member protein sequences were mined in FlyBase and multiply aligned using Clustal Omega (Sievers *et al.* 2011). To determine the best model of protein evolution for our data, we entered the alignment into ProtTest v 2.4. The appropriate substitution matrix was selected from the Akaike information criterion and Bayesian information criterion scores (Abascal *et al.* 2005; Darriba *et al.* 2011; Drummond and Strimmer 2001; Guindon and Gascuel 2003). Phylogenetic analysis was then completed using a maximum likelihood approach and rapid bootstrapping algorithm within RAxML v 7.2.8 Black Box (Stamatakis 2006; Stamatakis *et al.* 2008), on the Cipres web portal (Miller *et al.* 2010). Visualizations of the bipartition files were made using FigTree v 1.3.1 (http://tree.bio.ed.ac.uk/software/figtree/).

### Expression of *ppk* genes

Expression patterns of each member of the *ppk* gene family across different fly tissues were mined from FlyAtlas (Chintapalli *et al.* 2007). Microarray expression data from four independent microarrays were normalized and then graphed according to the expression level in different tissues. Temporal expression patterns of the *ppk* gene family were extracted from the modENCODE RNA-sequencing database (Celniker *et al.* 2009; Graveley *et al.* 2011). Normalized maximum expression was represented at different developmental stages, from the embryo to the adult fly in both males and females. To observe the spatial expression patterns of *ppk* and *ppk26* at a single cell resolution, we used the UAS-GAL4 binary expression system (Brand and Perrimon 1993) to express a membrane tethered version of EGFP (UAS-mCD8::GFP) using a previously published *ppk*-GAL4 line and a new *ppk26*-GAL4 line we have generated. *ppk*-GAL4 line was obtained from the Bloomington Drosophila Stock Center (stock no. 32078). The *ppk26*-GAL4 line was produced by amplifying a 2.2-kb fragment that included the first intron as well as sequences upstream of *ppk26* transcriptional start site (coordinates were 3L: 7447230-7449432 in release 5.47 of the *Drosophila* genome)

### PPK protein structure modeling

There are currently seven different accession numbers for structural models of DEG/ENaC channels in the PDB database, all which are based on the chicken acid-sensing ion channel (ASIC)1a protein. We chose to base our structural analyses of the *Drosophila ppk* gene family on the original 2QTS model (Jasti *et al.* 2007) because of the following reasons: (1) The 2QTS model has the best resolution (1.9 Å), which serves better as a template of homology modeling; and (2) 2QTS is a ligand-free model, which we predicted would work better as a modeling template since ASIC1a is a proton receptor, which is not necessarily a general property of DEG/ENaC channels. To generate structural predictions *in silico*, all PPK reference sequences and the template sequence (PDB ID: 2QTS) were aligned onto Hidden Markov model of amiloride-sensitive sodium channel family from PFAM [PFAM ID: PF00858(Punta *et al.* 2012)] by the program *hmmalign* in HMMER3 (Finn *et al.* 2011) and visualized by CLC Sequence Viewer. From the pair-wise sequence alignment of each PPK protein and the template, multiple structural models were generated by MODELER with default homology modeling protocol (Sali and Blundell 1993). The model with the best score was selected for further analysis. The molecular graphics software UCSF Chimera was used for structural visualization and analysis (Pettersen *et al.* 2004).

## Results and Discussion

### The *ppk* family in *Drosophila melanogaster*

The authors of previous studies have identified several DEG/ENaC family members, which were termed *pickpocket* (*ppk*) genes (Darboux *et al.* 1998; Liu *et al.* 2003a,b). However, a comprehensive scan of the fly genome for all family members has not been performed to date. We used a combination of current genome annotations as well as various homology search engines to identify 31 independent genes encoding for family members, which we named *ppk-ppk31* in complete agreement with prior annotations (Table 1).

**Table 1:** *ppk* genes identified in the *Drosophila melanogaster* genome

Name	Symbol	Alternative Name	CG No.	FB ID	Location
*pickpocket 1*	*ppk*	*ppk1*	*CG3478*	FBgn0020258	2L: 35B1-35B1
*ripped pocket*	*rpk*	*ppk2*	*CG1058*	FBgn0022981	3R: 82C5-82C5
*pickpocket 3*	*ppk3*		*CG30181*	FBgn0050181	2R: 59E3-59E3
*Nach*	*Nach*	*ppk4*	*CG8178*	FBgn0024319	2R: 53C14-53C14
*pickpocket 5*	*ppk5*		*CG33289*	FBgn0053289	3L: 78D5-78D5
*pickpocket 6*	*ppk6*		*CG11209*	FBgn0034489	2R: 56F11-56F11
*pickpocket 7*	*ppk7*		*CG9499*	FBgn0031802	2L: 26C3-26C3
*pickpocket 8*	*ppk8*		*CG32792*	FBgn0052792	X: 3D6-3D6
*pickpocket 9*	*ppk9*		*CG34369*	FBgn0085398	2R: 58A4-58A4
*pickpocket 10*	*ppk10*		*CG34042*	FBgn0065110	2L: 31E3-31E4
*pickpocket 11*	*ppk11*		*CG34058*	FBgn0065109	2L: 30C8-30C9
*pickpocket 12*	*ppk12*		*CG10972*	FBgn0034730	2R: 58E1-58E1
*pickpocket 13*	*ppk13*		*CG33508*	FBgn0053508	2L: 39A1-39A1
*pickpocket 14*	*ppk14*		*CG9501*	FBgn0031803	2L: 26C3-26C3
*pickpocket 15*	*ppk15*		*CG14239*	FBgn0039424	3R: 97B1-97B1
*pickpocket 16*	*ppk16*		*CG34059*	FBgn0065108	2L: 30C8-30C8
*pickpocket 17*	*ppk17*		*CG13278*	FBgn0032602	2L: 36A14-36A14
*pickpocket 18*	*ppk18*		*CG13120*	FBgn0032142	2L: 30C7-30C8
*pickpocket 19*	*ppk19*		*CG18287*	FBgn0039679	3R: 99B7-99B7
*pickpocket 20*	*ppk20*		*CG7577*	FBgn0039676	3R: 99B7-99B7
*pickpocket 21*	*ppk21*		*CG12048*	FBgn0039675	3R: 99B6-99B6
*pickpocket 22*	*ppk22*		*CG31105*	FBgn0051105	3R: 96B1-96B1
*pickpocket 23*	*ppk23*		*CG8527*	FBgn0030844	X: 16B4-16B4
*pickpocket 24*	*ppk24*		*CG15555*	FBgn0039839	3R: 100B9-100B9
*pickpocket 25*	*ppk25*	*lounge lizard (llz)*	*CG33349*	FBgn0053349	2R: 42E1-42E1
*pickpocket 26*	*ppk26*		*CG8546*	FBgn0035785	3L: 66A1-66A1
*pickpocket 27*	*ppk27*		*CG10858*	FBgn0035458	3L: 63E9-63E9
*pickpocket 28*	*ppk28*		*CG4805*	FBgn0030795	X: 15A9-15A10
*pickpocket 29*	*ppk29*		*CG13568*	FBgn0034965	2R: 60B6-60B6
*pickpocket 30*	*ppk30*		*CG18110*	FBgn0039677	3R: 99B7-99B7
*pickpocket 31*	*ppk31*		*CG31065*	FBgn0051065	3R: 97E5-97E6

**Figure 2:**
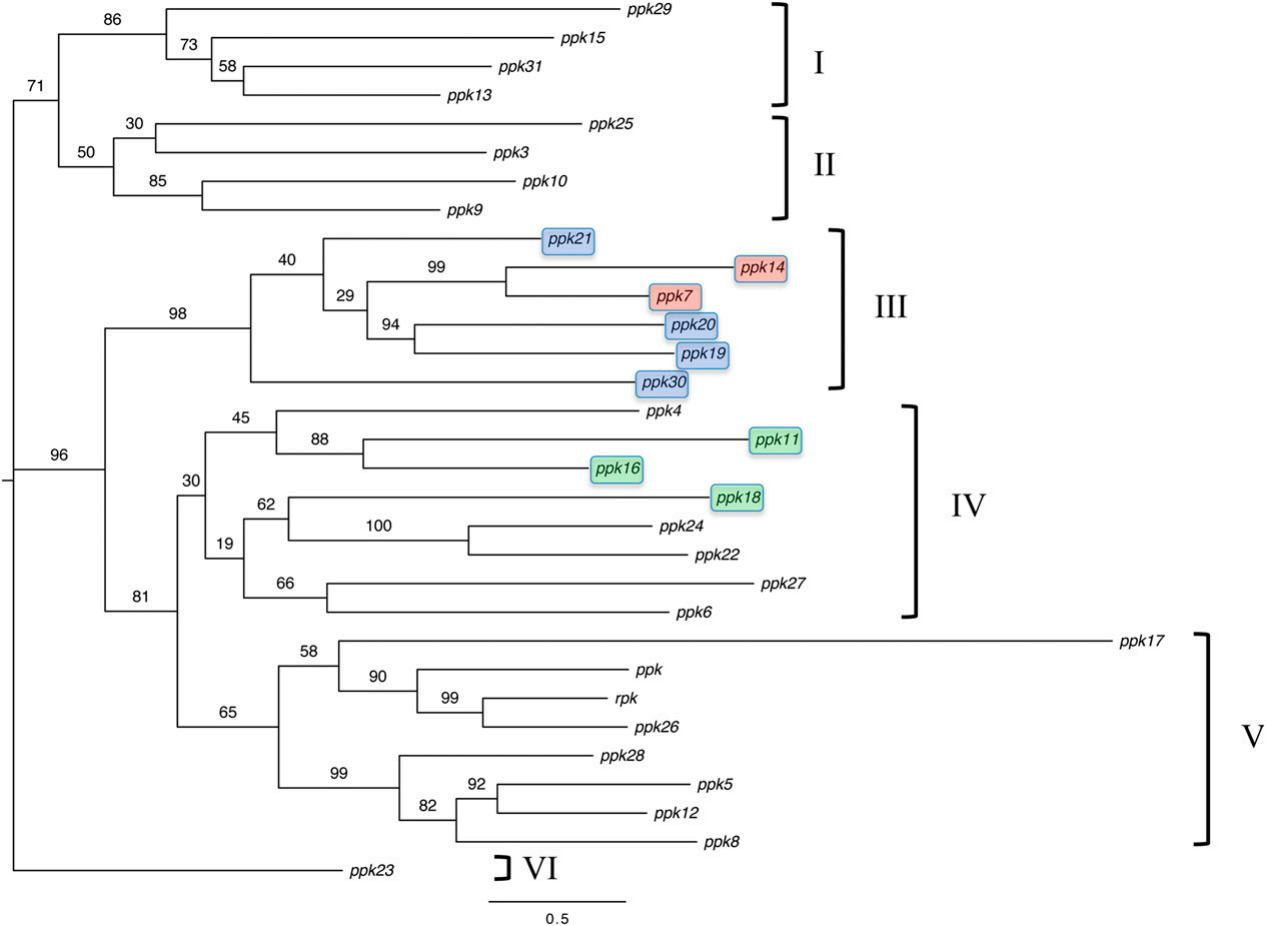
Maximum-likelihood unrooted phylogenetic tree inferred from multiply aligned amino acid sequences for *D. melanogaster* DEG/ENaC *ppk* genes. A total of 31 DEG/ENaC amino acid sequences are divided into six clusters and labeled as groups I-VI. Bootstrap values are given on branches and amino acid substitution rate is given at the bottom of the figure. Colors represent chromosomally clustered subunits (see Figure 5 for details).

Alignment of all identified PPK sequences revealed a highly conserved cysteine-enriched domain, which contains five disulfide bonds by 10 highly conserved cysteines in the thumb domain (Figure 1, A and B). Unrooted protein phylogenetic analysis of all identified *ppk* genes in the *D. melanogaster* genome indicated that this protein family is composed of at least six distinct subfamilies (labeled as I-VI; Figure 2). Overall, the relationship between *ppk* genes in subfamilies III, IV, and V are well resolved and supported by high bootstrap values. However, few genes such as *ppk17* and *ppk23* are not well resolved in our phylogeny, despite multiple (N = 4) runs of the alignment and phylogenetic tree programs, which produced the same results for each run. The inability to resolve certain *ppk* relationships is likely due to the high amount of divergence in amino acid sequence between *ppk* family members (Supporting Information, Table S1).

### *ppk* genes are highly conserved in the *Drosophila* lineage

We subsequently extended our gene search analyses to the sequenced genomes of additional 11 *Drosophila* species as well as to the genome of *Anopheles gambiae* (African malaria mosquito), which served as a dipteran outgroup (Table S2) (Holt *et al.* 2002). These analyses revealed that the majority of the *D. melanogaster ppk* radiation is preserved in all 12 sequenced *Drosophila* genomes (Bhutkar *et al.* 2008; Singh *et al.* 2009), indicating *ppk* diversification occurred early in the evolution of the *Drosophila* lineage.

### Expression patterns, structural variations, and predictions of function

**Figure 3:**
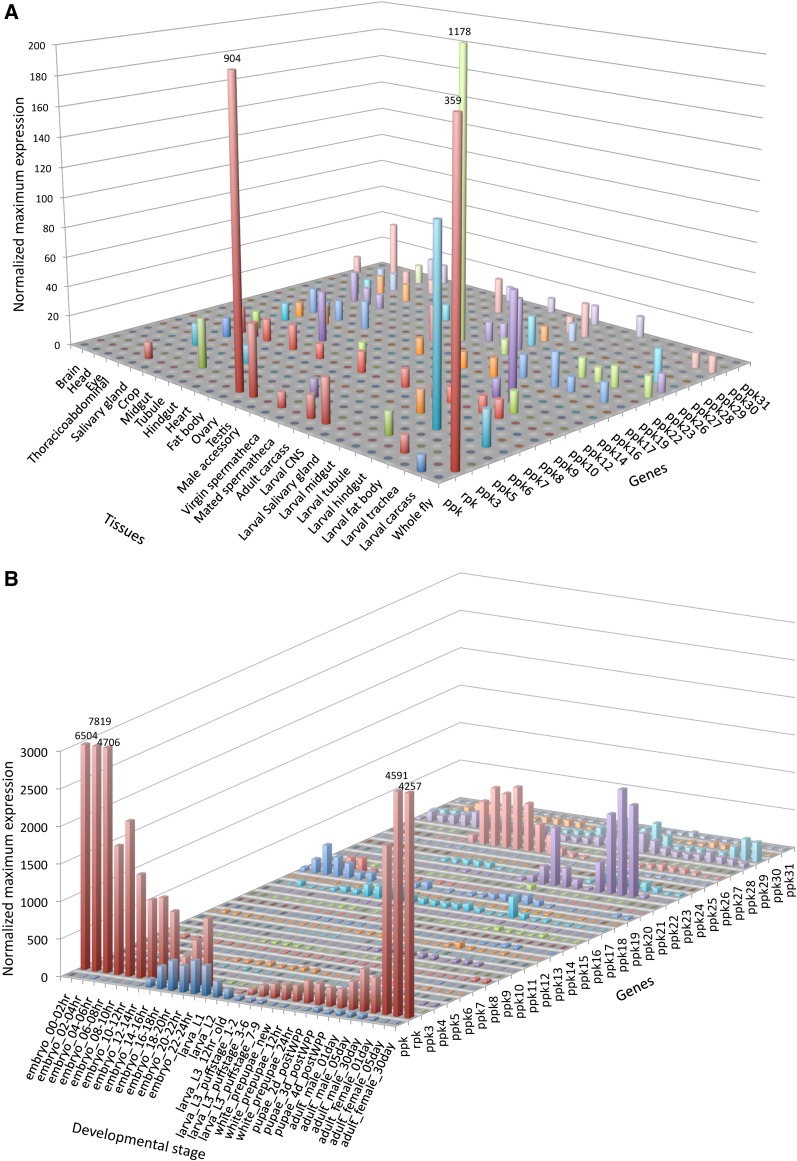
(A) Spatial expression patterns of *ppk* genes. Microarray expression data were extracted from FlyAtlas (Chintapalli *et al.* 2007). Expression represents the average signal from four independent microarrays. (B) Temporal expression patterns of *ppk* genes. Data were extracted from the modENCODE RNA-seq database (Celniker *et al.* 2009). Expression levels are represented as log_2_ values of the original coverage. Numbers at the tops of truncated bars show actual expression values.

**Figure 4:**
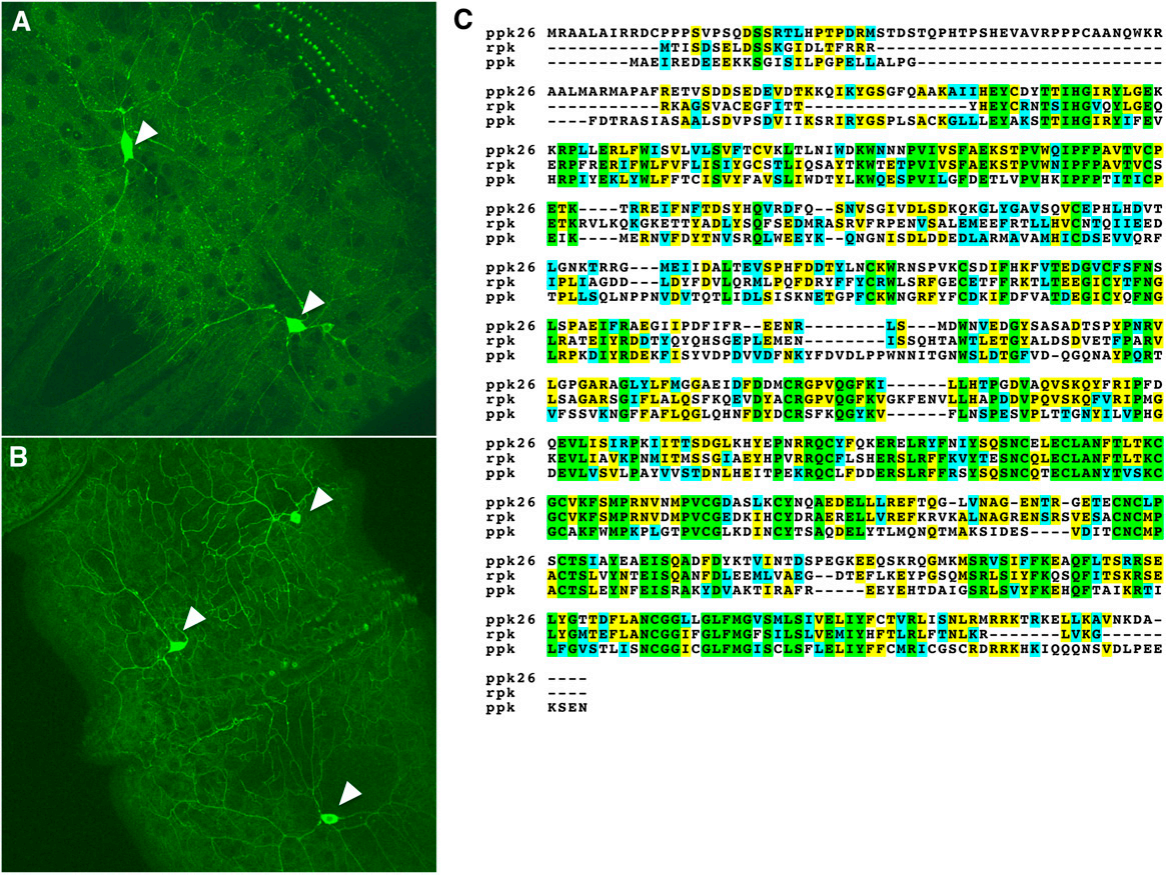
*ppk* and *ppk26* expression in larval multidendritic neurons. (A) *ppk-GAL4 x UAS-mCD8*::*GFP*. (B) *ppk26-GAL4 x UAS-mCD8*::*GFP*. White arrows indicate cell body. (C) Alignment of *ppk*, *rpk*, and *ppk26* amino acid sequence. Green, residues are conserved across all proteins examined; yellow, residues are conserved in some species; blue, conserved substitutions.

Analyses of mRNA expression levels across various *D. melanogaster* tissues (Figure 3A) and developmental stages (Figure 3B) indicated that individual *ppk* family members show different expression profiles in both mRNA expression level and temporal and spatial expression patterns. These data suggest that this family has evolved to serve a wide variety of physiological functions. Although a handful of subunits have been implicated in mechanosensation and chemosensory perception, the contribution of sequence variation to physiological function remains unclear. Of particular interest is subfamily V, which includes the *ppk*, *rpk*, and *ppk26* cluster (Figures 2 and 4). Both *rpk* and *ppk* have been implicated in mechanosensation in larvae, although in different types of multidendritic neurons, and are likely to have similar but independent functions in neurons (Adams *et al.* 1998; Kim *et al.* 2012; Tsubouchi *et al.* 2012; Zhong *et al.* 2010). The spatial expression pattern of *ppk26*, which is a close paralogue of the *ppk* and *rpk* subunits is very similar to *ppk* suggesting the two subunits might be co-expressed (Figure 3A). To further explore this, we generated a transgenic *Drosophila* line that can report the expression patterns of the gene using the UAS-GAL4 system (Brand and Perrimon 1993). As predicted by the mRNA expression data, the expression of the *ppk26* gene is enriched in class IV multidendritic sensory neurons, which also express *ppk* (Figure 4). These data suggest that *ppk26* and *ppk* are either redundant or are corequired for some aspect of mechanosensation in these nociceptive neurons. In sum, though the functions of all DEG/ENaC subunits are not yet known, we hypothesize that *ppk*, *rpk*, and *ppk26*, which show sequence and structural similarities and are expressed in multidendritic neurons, may have similar functions in nociceptive mechanosensation.

### Subfamily III is not present in mosquitoes

As expected, *ppk* family gene conservation between the *D. melanogaster* and the mosquito genomes was lower than across the *Drosophila* lineage (Table S2). We identified only 18 family members in the genome of *A. gambiae*, of which 17 had homologs in the *Drosophila* genome and one that seemed to be a mosquito-specific subunit (AGAP006704; Table S2). These data suggest that the extreme diversity we observed in the *Drosophila* lineage is not shared by all dipteran species.

Closer examination of the conservation of *Drosophila ppk* subfamilies in *A. gambiae* revealed that none of the genes represented in subfamily III was present in the mosquito genome, suggesting this subfamily is not common in all dipteran species. (Figure 2 and Table S2). In contrast, we have indentified at least one homologous gene from each of the remaining *ppk* subfamilies in the mosquito genome (Table S2). These data may suggest that each *ppk* subfamily (with the exception subfamily III) represents a core DEG/ENaC physiological function in Diptera.

### Diversity, duplications, gene syntenies, and sequence homologies

Examination of overall gene conservation across all sequenced *Drosophila* species indicated that protein phylogeny followed closely the predicted species phylogeny (Clark *et al.* 2007). We examined in more detail several subfamilies of conserved *ppk* genes across the 12 sequenced *Drosophila* genomes as well as the malaria mosquito *A. gambiae*. We first examined the highly conserved subgroup that included *ppk*, *rpk*, and *ppk26*. All three genes are highly conserved across all 12 genomes (Table S2).

Although each *Drosophila* genome includes one subunit that corresponds most closely to *ppk*, *rpk*, or *ppk26*, the mosquito genome encodes four related subunits, all of which are clustered with the *Drosophila ppk26* (Table S2). These data suggest that *ppk26* represents an early dipteran subunit, which may have independently diversified in the *Drosophila* and mosquito lineages.

**Figure 5:**
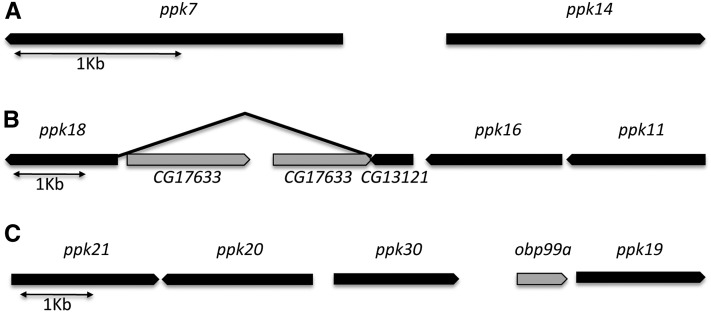
Chromosomal clusters of *ppk* genes. (A) Cluster of *ppk7* and *ppk14* located at 2L: 26C3-26C3. (B) Cluster of *ppk18*, *ppk16*, and *ppk11* located at 2L: 30C8-30C9. Note that although CG13121 is currently annotated as a separate gene, molecular analyses of mRNA clones indicate that it is part of the *ppk18* locus (not shown). (C) Cluster of *ppk21*, *ppk20*, *ppk30*, and *ppk19* located at 3R: 99B6-99B7. Black boxes, *ppk* genes; gray boxes, none-*ppk* genes.

Nine of the 31 *ppk* genes we have identified in the *D. melanogaster* genome are chromosomally clustered (Figure 5). Protein phylogeny indicated that the majority of genomic clusters were likely the result of gene duplications since the clustered genes showed high sequence similarities and belonged to the same *ppk* subfamilies (Boxed genes names in Figure 2). An exception is *ppk18*, which is clustered with *ppk11* and *ppk16* (Figure 5B), two less related subunits (Figure 2). These data suggest that the clustering of these three subunits might have been the result of selection underlying shared physiological and/or cellular functions. *ppk11* has been implicated in salt taste (Liu *et al.* 2003b). We speculate that these three subunits might contribute to salt taste in *Drosophila* by forming the sodium sensitive ion channel. (Adams *et al.* 1997; Chandrashekar *et al.* 2006; Chandrashekar *et al.* 2010; McDonald *et al.* 1995; Snyder *et al.* 1995). We found that all identified *D. melanogaster ppk* genomic clusters are conserved across all 12 *Drosophila* species genomes (not shown), indicating that the molecular events that led to clusters formation happened early in the species radiation of the *Drosophila* genus.

**Figure 6:**
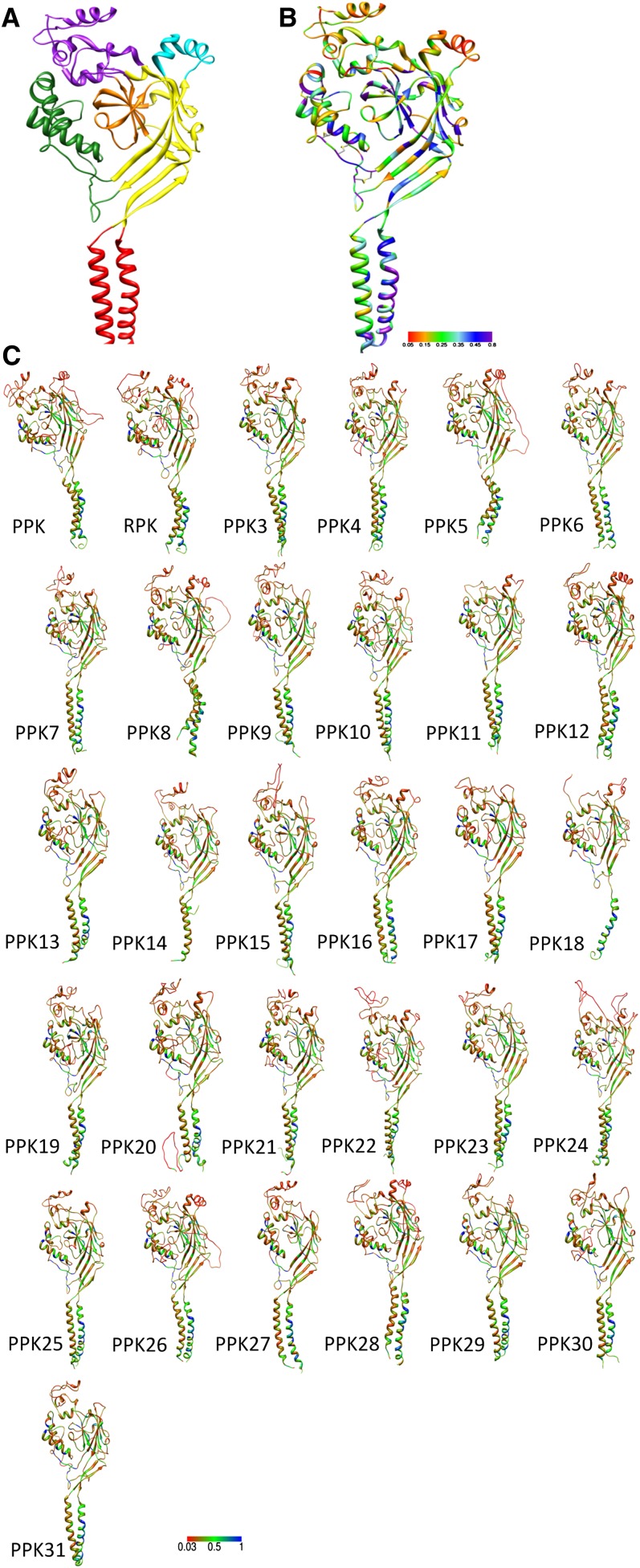
Structural modeling of the *ppk* family in *Drosophila*. (A) Domain organization of the chicken ASIC1a subunit (Jasti *et al.* 2007) Red: TM1 (left helix), TM2 (right helix); yellow: Palm; cyan: Knuckle; orange: beta-ball; purple: Finger; green: Thumb. (B) ASIC1a subunit rendered by conservation information from its alignment with the *ppk* family. The regions colored in purple are highly conserved residues, whereas those colored in red are most variable in the alignment. (C) Predicted structure for all *Drosophila* PPK subunits. The rainbow scale represents the residue conservation scores. The regions colored in red are most variable whereas regions in blue are highly conserved.

In addition to linear protein sequence analyses, we also built structural models of all PPK proteins by using the published crystal structure of the chicken ASIC (Jasti *et al.* 2007) as a guide. According to the protein conservation information from multiple alignment of the *ppk* family, we rendered a general *Drosophila* PPK model (Figure 6A). Furthermore, we used the resolved ASIC structure to predict structural models for all individual *Drosophila ppk* subunits (Figure 6B). Close inspection of the structure and the overall protein alignment revealed 10 highly conserved cysteines (>90% conservation), which are likely to form up to five disulfide bonds.

**Figure 7:**
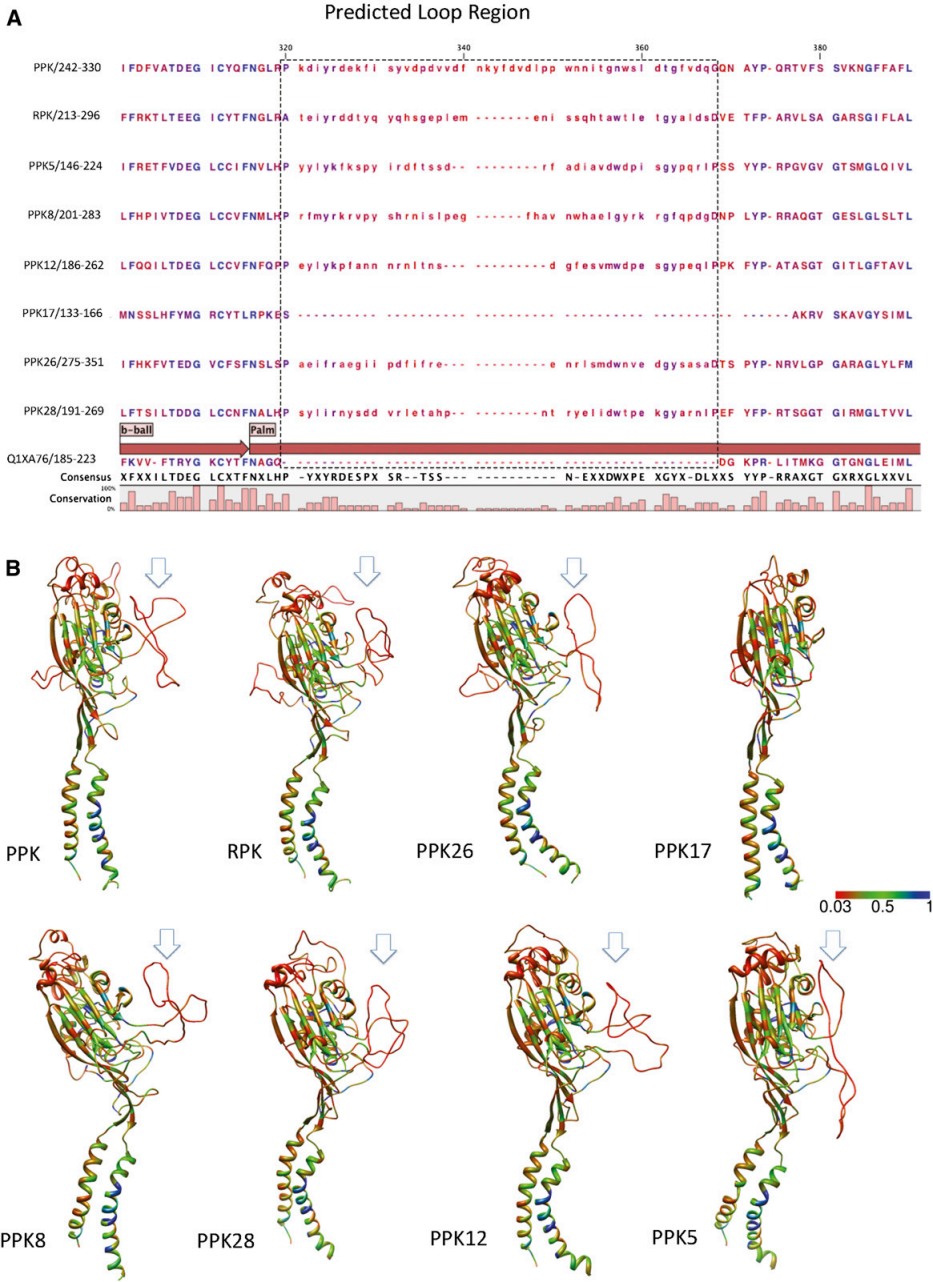
(A) The alignment of individual subunits from *ppk* subfamily Group V (for full Group V alignment, see Figure S1). The dashed frame marks the unstructured loop region. Note that PPK17 does not have the unstructured loop region. Q1XA76 is the chicken ASIC Uniprot Accession ID. Consensus sequence was built from the majority of the aligned residues. The bars in the bottom represent conservation percentage after alignment. (B) Unstructured loop region in the subfamily Group V. Predicted structures for all *D. melanogaster* PPK subunits are shown in Figure 6. The rainbow scale represents residue conservation as in Figure 6.

We also found that most family members from group V (Figure 2) have a long unstructured loop without a matched structural template in the resolved vertebrate model (Figure 7, with the exception of PPK17). Whether this unstructured loop plays a functional role is unknown. However, *ppk* is expressed in type IV multidendritic neurons, which play a role in thermal and mechanical nociception in fly larvae (Adams *et al.* 1998; Ainsley *et al.* 2003; Hwang *et al.* 2007; Kim *et al.* 2012; Zhong *et al.* 2010). The recent publication, which implicates *rpk* in mechanosensotive functions in Class III multidendritic neurons, and our finding that *ppk26* is expressed in Class IV multidendritic neurons in a similar pattern to *ppk* suggest that other members of this cluster might be playing similar roles in mechanotransduction pathways. Further, our data raise the intriguing hypothesis that the large unstructured side loop that is a signature of cluster V may be playing a role in mechanosensory functions, possibly by interacting with extracellular matrix proteins (Arnadottir and Chalfie 2010; Arnadottir *et al.* 2011; Brown *et al.* 2008; Chalfie 2009; Geffeney *et al.* 2011; Huber *et al.* 2006; Zhang *et al.* 2004).

Here we show a comprehensive analysis of an emerging and important family of ion channels in the genetically tractable fruit fly model. As the importance of the DEG/ENaC family continues to increase, studies in *Drosophila* could reveal novel insights into the physiological functions of this enigmatic group of ion channels. Taking advantage of the wealth of genetic and evolutionary data in the *Drosophila* group as well as other insect species, we intend to generate novel testable structure-function hypotheses that would likely shed additional light on the physiological functions of these proteins in species ranging from the worm to humans.

## Supplementary Material

Supporting Information
