# Supplementary material for: The Genetic Architecture of Degenerin/Epithelial Sodium Channels in Drosophila
Source: G3 (Bethesda). 2013 Mar 1;3(3):441–50. doi: 10.1534/g3.112.005272 (PMC3583452; doi:10.1534/g3.112.005272)
Supplement: Supporting Information [file supp_3_3_441__index.html]

Supporting Information 

# The Genetic Architecture of Degenerin/Epithelial Sodium Channels in *Drosophila*

## Supporting Information for Zelle *et al.*, 2013

**Files in this Data Supplement:**

- Supporting Information - Figure S1 and Tables S1 and S2 (PDF, 141 KB)
- Figure S1 - Complete protein alignment of PPK proteins from group V (PDF, 423 KB)
- Table S2 - *ppk* genes identified in sequenced *Drosophila* genomes (PDF, 105 KB)
- Table S1 - Pair wise % amino acid identities across the complete ppk protein family in *Drosophila melanogaster* (PDF, 47 KB)
