# Supplementary material for: The Genetic Architecture of Degenerin/Epithelial Sodium Channels in Drosophila
Source: G3 (Bethesda). 2013 Mar 1;3(3):441–50. doi: 10.1534/g3.112.005272 (PMC3583452; doi:10.1534/g3.112.005272)
Supplement: Supporting Information [file supp_3.3.441_005272SI.pdf]

G3 Genes | Genomes | Genetics

The Genetic Architecture of Degenerin/Epithelial Sodium Channels in *Drosophila*

Kathleen M. Zelle[1,2], Beika Lu[1,2,3], Sarah C. Pyfrom[1], and Yehuda Ben-Shahar[1]*

1. Department of Biology, Washington University in St. Louis, MO 63130, USA
2. Equal contribution.
3. Current address: School of Life Sciences and Technology, Tongji University, Shanghai 200092, China

Corresponding author:
Yehuda Ben-Shahar
Department of Biology
Washington University in St. Louis
One Brookings Dr., St. Louis
MO 63130
benshahary@wustl.edu
Tel: (314) 935-3484
Fax: (314) 935-4432

DOI: 10.1534/g3.112.005272

Figure S1 Complete protein alignment of PPK proteins from group V. All reference protein sequences for PPK proteins from group V (Fig. 2) were aligned to the reference chicken ASIC1 protein (Q1XA76). Bars below alignment represent % conservation for each amino acid in the reference sequence. Identified protein domains from the crystal structure of the chicken ASIC1 proteins are marked as solid arrows above the reference sequence. Conserved residues are in uppercase. Font shades represent level of conservation going from dark blue for complete conservation to light red.

Figure S1 is available for download at http://www.g3journal.org/lookup/suppl/doi:10.1534/g3.112.005272/-/DC1.

Table S1 Pair wise % amino acid identities across the complete ppk protein family in *Drosophila melanogaster.*

Table S1 is available for download at http://www.g3journal.org/lookup/suppl/doi:10.1534/g3.112.005272/-/DC1.

Table S2 ***ppk* genes identified in sequenced *Drosophila* genomes.** *Dmel, Drosophila melanogaster; Dsec, D. sechellia; Dsim, D. simulans; Dyak, D. yakuba; Dere, D. erecta; Dana, D. ananassae; Dpse, D. pseudoobscura, Dper, D. persimilis; Dwil, D. willistoni; Dmoj, D. mojavensis; Dgri, D. grimshawi; Dvir, D. virilis; Agam, Anopheles gambiae*. X, no homologous proteins were identified; Y, TBLASTN search revealed un-annotated homologous sequences.

Dmel	*Dsec*	*Dsim*	*Dyak*	*Dere*	*Dana*	*Dpse*	*Dper*	*Dwil*	*Dmoj*	*Dgri*	*Dvir*	*Agam*
ppk	GM14849	GD22009	GE25026	GG24259	GF15015	GA17471	GL25771	GK18092	GI20631/GI20633	GH20995	GJ21600	X
rpk	GM10769	GD19743	GE25429	GG12538	GF18905	GA10410	GL21653	GK11507	X	GH12436	GJ19160	X
ppk3	GM15534	GD25038	GE11559	GG20022	GF11840	GA15705	GL17553	GK23048	GI19625	GH20442	GJ14993	AGAP006720
NaCh	GM20042	GD25525	GE14047	GG22253	Nach	GA20871	GL11823	GK10729	GI18494	GH22707	Nach	X
ppk5	GM22378	GD14969	GE19769/ GE19766	GG16195	GF23723	GA23355	GL11889	Y	GI13694	GH16657	GJ11315	X
ppk6	GM16400/ ppk6	ppk6	GE13818	GG20878	GF11044	GA24082	GL11802	Y	GI18368	GH20930	GJ21439	AGAP010430
ppk7	GM17947	GD22586	GE18451	GG23630	GF15394	GA21835	GL26268	GK15363	GI17506	GH10991	GJ15216	X
ppk8	GM12297	Y	GE16304	GG18660	GF22642	GA17142	GL15946	GK16264	GI15707	X	X	X
ppk9	GM15874+ GM15873	GD11635	GE12231+ GE12230	GG22150+ GG22149	GF13278	GA12323	GL16963	GK15936	GI18741	GH21139	GJ21761	AGAP004474
ppk10	GM11655	GD22255	GE13615	GG10385	GF14095	Y	GL19139	GK14883	GI20510	GH13616	GJ13850	AGAP009789
ppk11	GM12254	GD22335	GE10314	GG24006	GF21875	GA25876	GL19208	GK24817	GI18203	GH25067	GJ14732	X
ppk12	GM15912	GD11671	GE14185	GG22192	GF11893	GA10679	GL17548	GK20821	GI20562	GH21367	GJ22416	X
ppk13	GM23280	GD21656	GE13005	GG21527	GF24164	GA25474	GL25869	GK21999	GI17841	GH11334	GJ17335	AGAP007945
ppk14	GM18622	GD23404	GE13813	GG10406	GF11173	GA21837	GL25522	GK14756	GI11553	GH10745	GJ12828	X
ppk15	GM10186	GD18138	GE10633	GG12190	GF20729	GA12851	GL21999	GK13313	GI10129	GH18177	GJ23349	AGAP008378
ppk16	GM12265	GD22336	GE10325	GG24007	GF21878	GA25877	GL19209	GK24818	GI18204	GH25068	GJ14742	AGAP009590
ppk17	GM17158	GD21897	GE13164	GG20108	GF14545	GA12165	GL19333	GK18158	GI14930	GH10205	GJ24072	AGAP010146
ppk18	GM12287	GD22338	GE10347	GG24009	GF21883	GA12059	GL19211	GK24820	GI18206	GH25070	GJ14764	X
ppk19	GM12801	GD21448	GE23866	GG11677	GF23358	GA14875	GL13899	GK11909/ GK11911	GI23407	GH18908	GJ10613	X
ppk20	GM12234	Y	GE10443	Y	GF22877	GA20451	Y	GK11175	GI24197	GH18687	GJ10545	X
ppk21	GM12798	GD21445	GE23863	GG11673	GF23355	GA11359	GL13896	GK11905	GI23122	GH18904	GJ10610	X
ppk22	GM23453	GD18259	GE10766	GG12311	GF18007	GA16013	GL13559	GK22757	GI22199	GH16423	GJ24318	X
ppk23	GM13310	GD24489	GE15587	GG18177	GF22609	GA21139	Y	GK19918	GI14637	GH12006	GJ19288	AGAP000840
ppk24	GM12934	GD21571	GE10931	GG11801	GF17779	GA27013	GL13740	GK22758	GI22200	GH16433	GJ24319	AGAP001631
ppk25	GM20909	GD10437+ GD10438	GE19087	GG23236	GF13151	GA24620	GL11130	GK21769	GI19722	GH22016	GJ17577	AGAP005516
ppk26	GM14846	Y	GE21620	GG14431	GF10647	GA21154	GL26473	GK17385	GI12863	GH14886	GJ13005	AGAP011610/ AGAP011611/ AGAP012279/ AGAP010967
ppk27	GM14042	GD13321	GE18017	GG14248	Y	Y	GL15374	GK19159	GI12811	Y	Y	X
ppk28	GM13471	GD17312	GE17629	GG19083	GF21749	GA18445	Y	GK25370	GI15345	GH11924	GJ19317	AGAP001602
ppk29	GM18277	GD24975	GE11489	GG19957	Y	X	GL10426	GK21926	X	X	X	X
ppk30	GM12799	GD21446	GE23864	GG11675	GF23356	GA14800	GL13897	GK11906/ GK11907	GI23405	GH18906	GJ10611	X
ppk31	GM10375	Y	Y	GG11534	GF23284	GA15980	GL23907	GK11886	GI22270	GH19633	GJ24060	AGAP000657
X	X	X	X	X	X	X	X	X	X	X	X	AGAP006704
