# Supplementary figures and images for: The Genetic Architecture of Degenerin/Epithelial Sodium Channels in Drosophila
Source: G3 (Bethesda). 2013 Mar 1;3(3):441–50. doi: 10.1534/g3.112.005272 (PMC3583452; doi:10.1534/g3.112.005272)

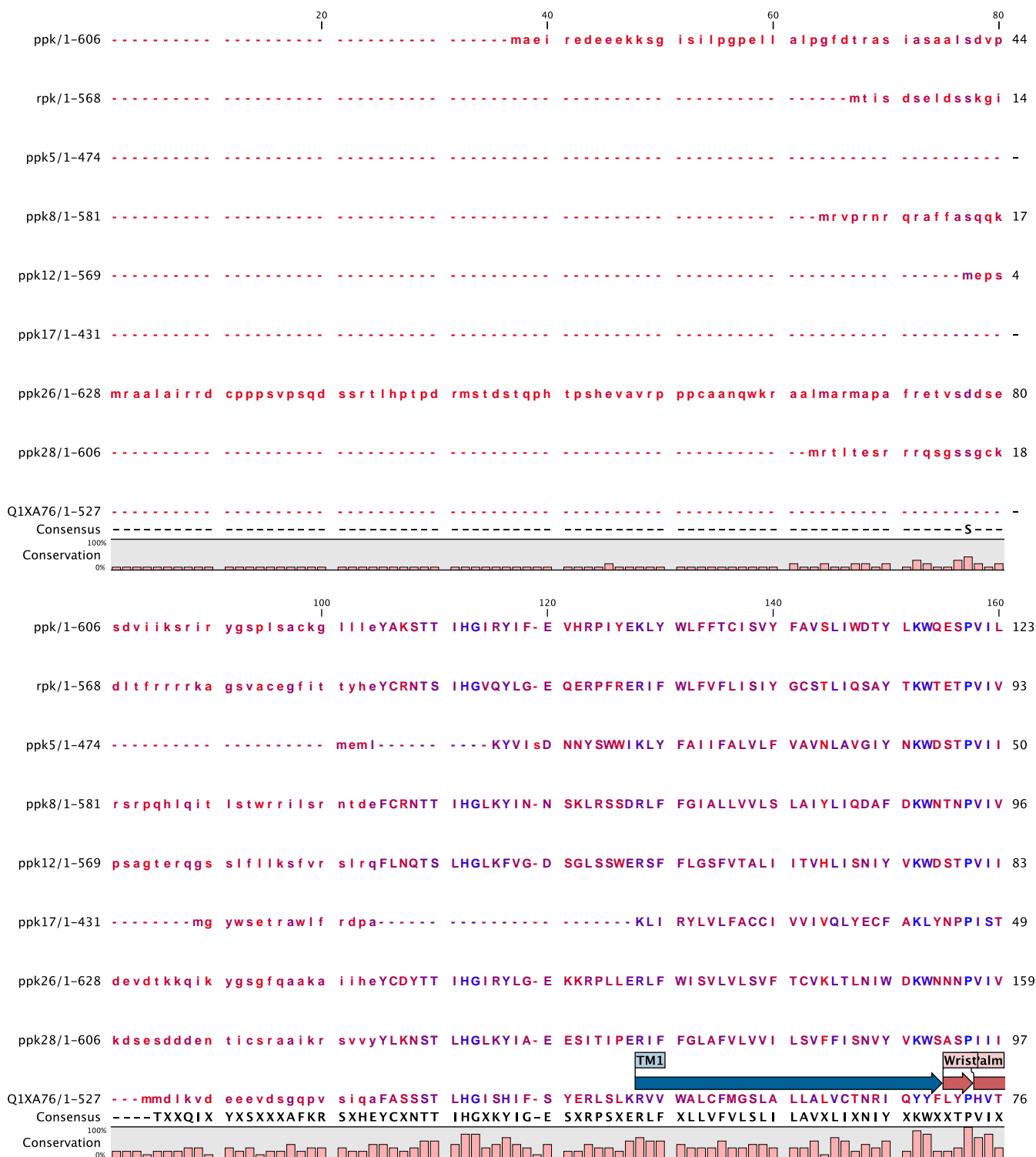

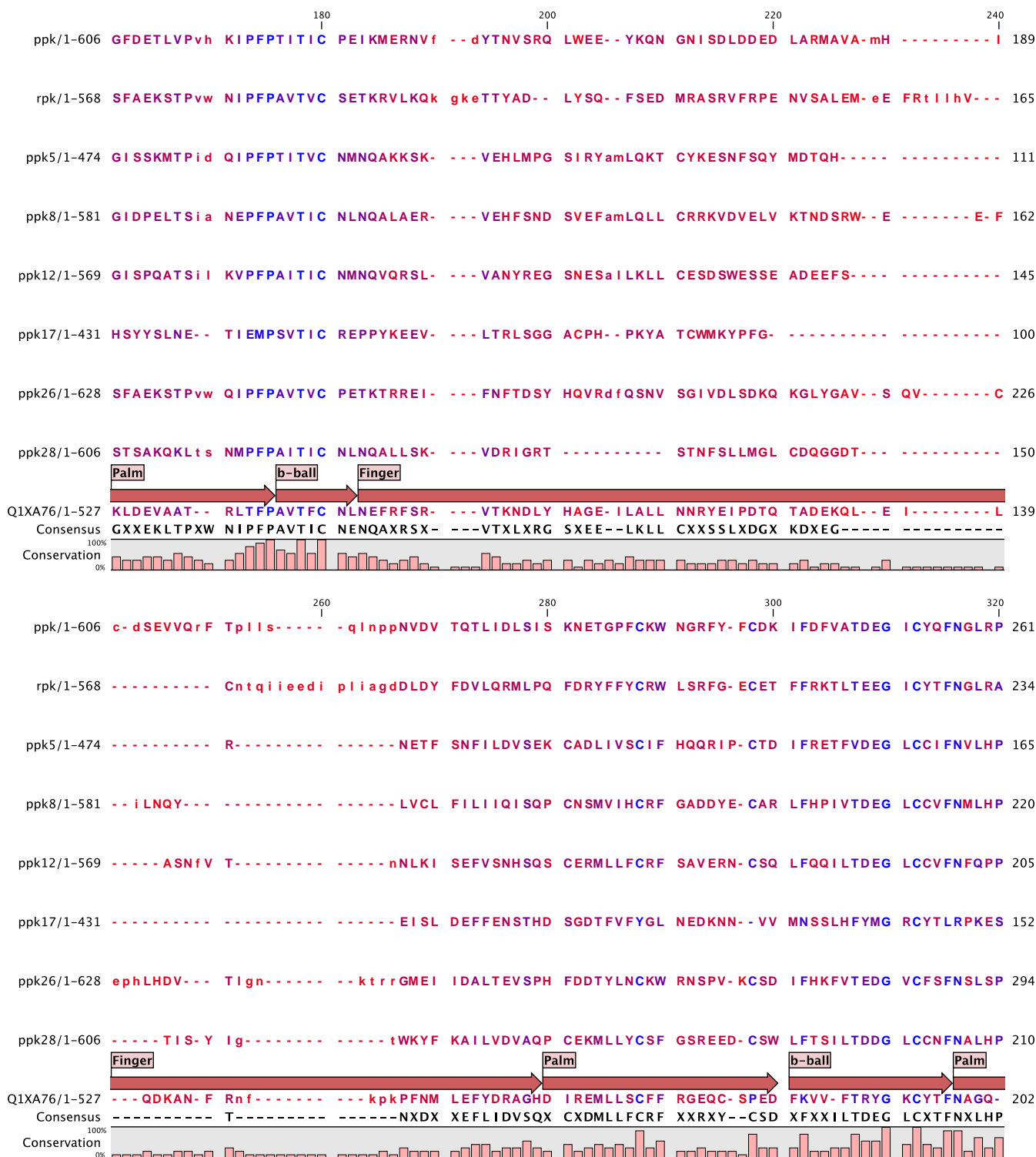

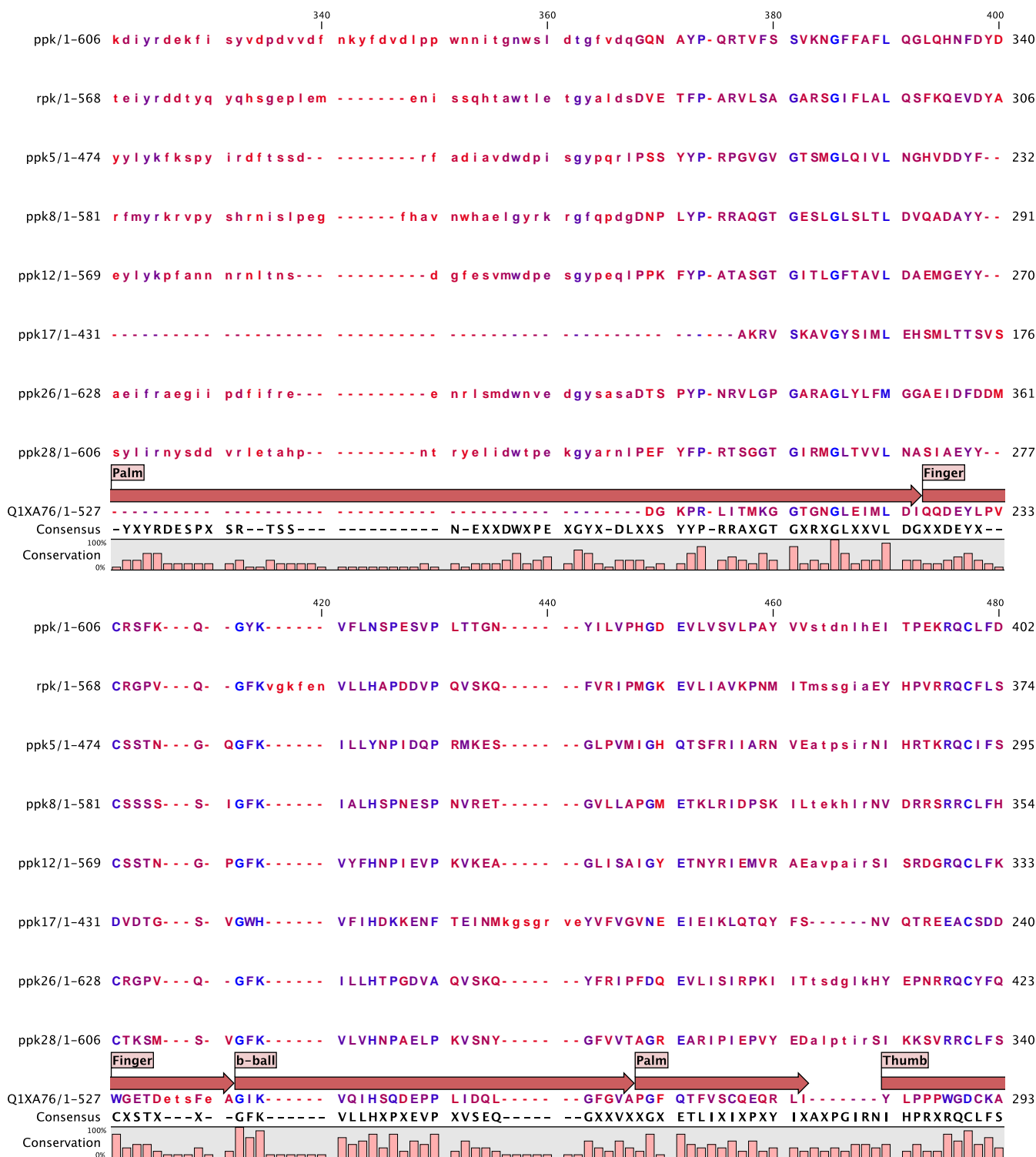

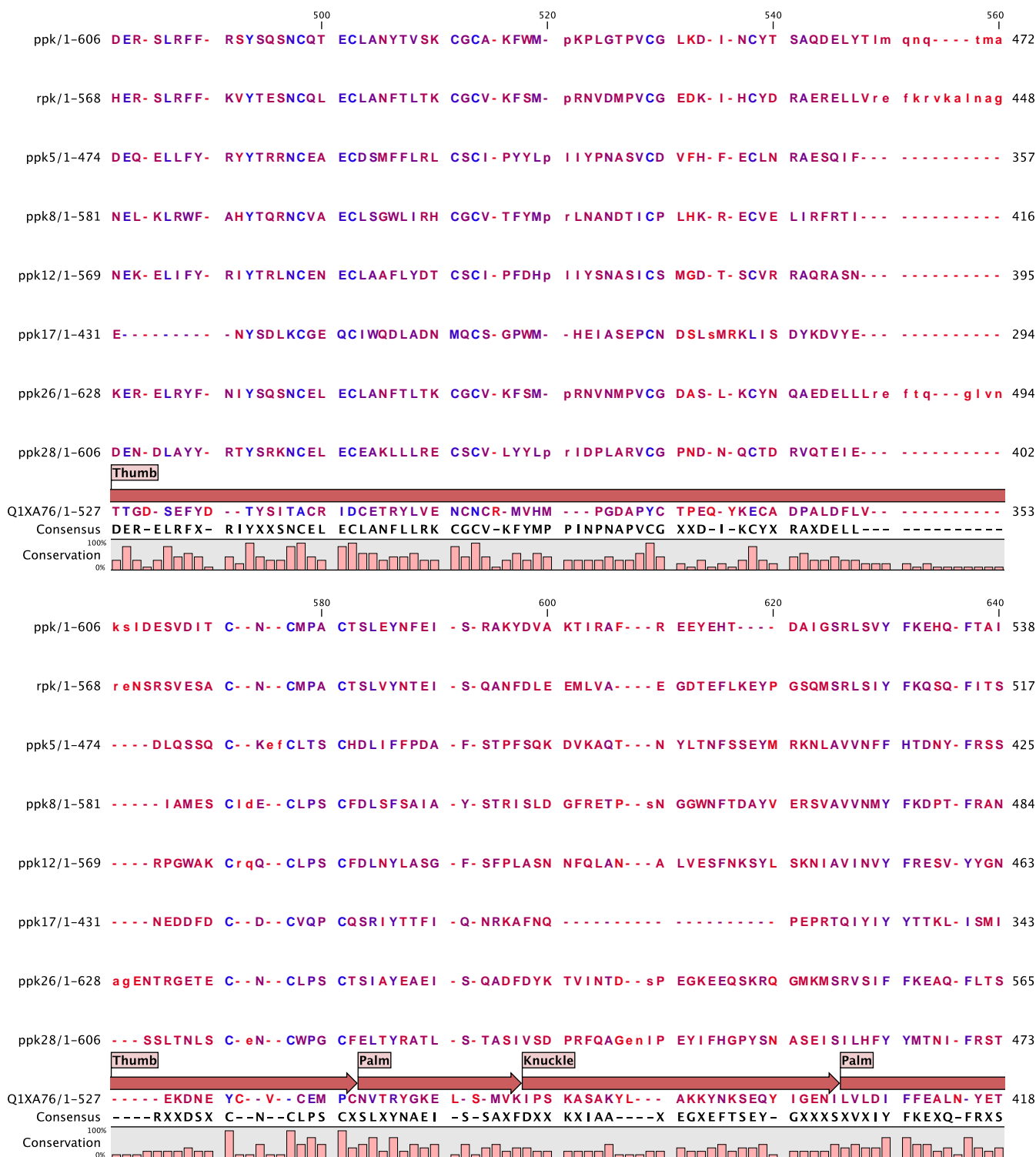

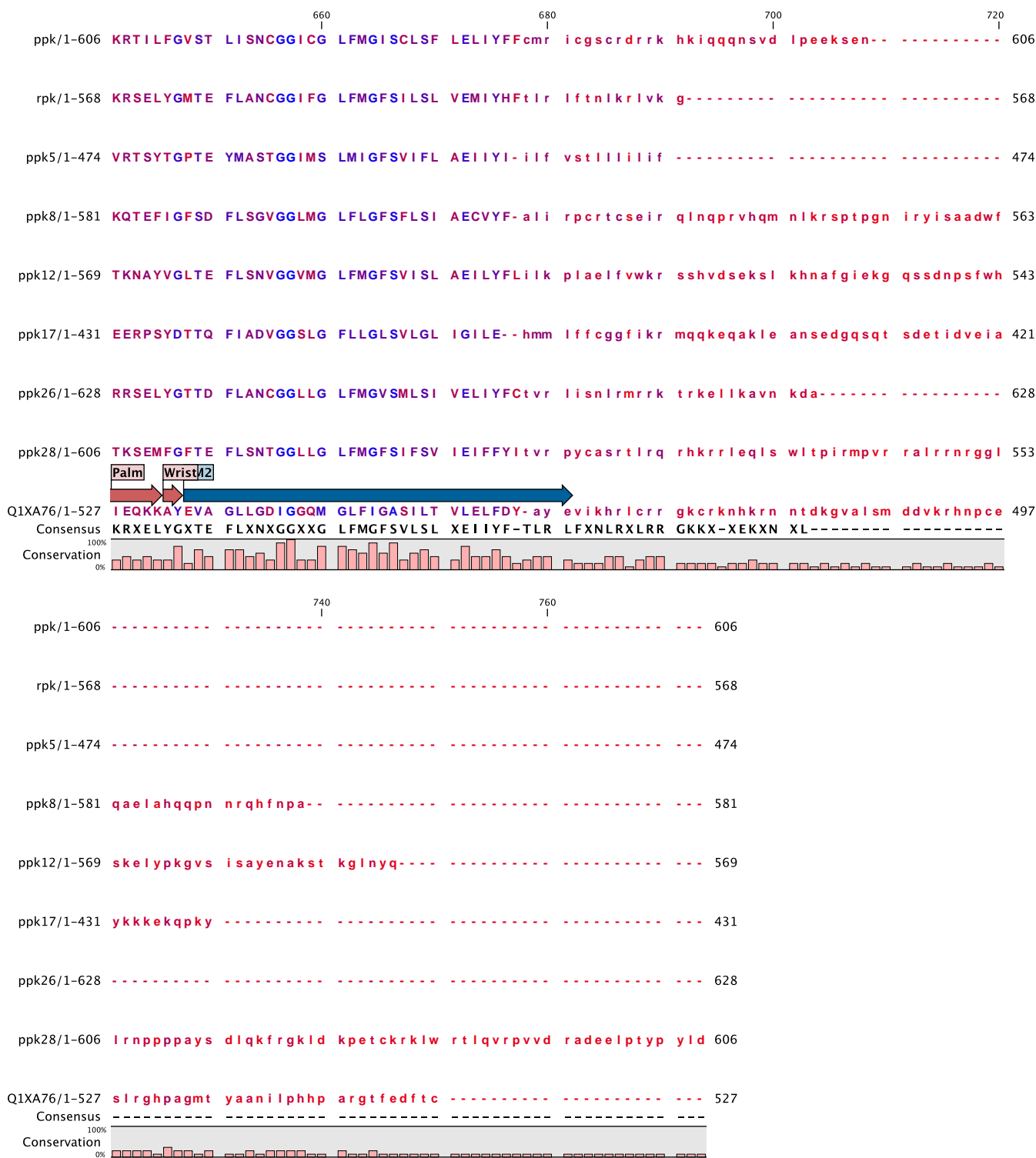

Supplement: Supporting Information [file supp_3.3.441_FigureS1.pdf]
